# A Randomized Controlled Trial Evaluating a Low-Volume PEG Solution Plus Ascorbic Acid versus Standard PEG Solution in Bowel Preparation for Colonoscopy

**DOI:** 10.1155/2015/326581

**Published:** 2015-11-15

**Authors:** Masahiro Tajika, Tsutomu Tanaka, Makoto Ishihara, Nobumasa Mizuno, Kazuo Hara, Susumu Hijioka, Hiroshi Imaoka, Takamitsu Sato, Tatsuji Yogi, Hideharu Tsutsumi, Toshihisa Fujiyoshi, Nobuhiro Hieda, Nozomi Okuno, Tsukasa Yoshida, Vikram Bhatia, Yasushi Yatabe, Kenji Yamao, Yasumasa Niwa

**Affiliations:** ^1^Department of Endoscopy, Aichi Cancer Center Hospital, 1-1 Kanokoden, Chikusa-ku, Nagoya, Aichi 464-8681, Japan; ^2^Department of Gastroenterology, Aichi Cancer Center Hospital, 1-1 Kanokoden, Chikusa-ku, Nagoya, Aichi 464-8681, Japan; ^3^Department of Medical Hepatology, Institute of Liver and Biliary Sciences, D-1, Vasant Kunj, New Delhi, Delhi 110070, India; ^4^Department of Pathology and Molecular Diagnosis, Aichi Cancer Center Hospital, 1-1 Kanokoden, Chikusa-ku, Nagoya, Aichi 464-8681, Japan

## Abstract

Evaluation of polyethylene glycol electrolyte lavage solution containing ascorbic acid (PEG-ASC) has been controversial in the point of its hyperosmolarity, especially in old population. So we therefore designed the present study to compare the efficacy, acceptability, tolerability, and safety of 1.5 L PEG+ASC and 2 L standard PEG electrolyte solution (PEG-ELS), not only in the general population, but also in patients of advanced age. Randomization was stratified by age (<70 years or 70> years), and hematological and biochemical parameters were compared in each age group, especially with respect to the safety profile of each regimen. As a result, the 1.5-L PEG-ASC regimen had higher patient acceptability than the 2-L PEG-ELS regimen. Tolerability, bowel cleansing, and safety were similar between regimens. However, we demonstrated significant statistical changes in the hematological and biochemical parameters after taking bowel preparation solutions, not only in the PEG+ASC group, but also in the PEG-ELS group. No significant differences in the safety profile were found between subjects aged less than 70 years and those aged 70 years or more; nevertheless, regardless of age, proper hydration is needed throughout the bowel preparation process.

## 1. Introduction

Polyethylene glycol electrolyte lavage solution (PEG-ELS) is the gold standard for bowel cleansing (strong recommendation, high quality) for colonoscopy by recommendation of the US Multi-Society Task Force on Colorectal Cancer [1] (American College of Gastroenterology, American Gastroenterological Association, and American Society of Gastrointestinal Endoscopy). However, although osmotically balanced electrolyte lavage solutions offer safe and effective cleansing [2–4], volume-related discomfort and adverse experiences have decreased the percentage of patients completing the preexamination preparation. This is mainly due to large volumes of fluid required for bowel preparation, unpleasant taste, and an increase in the incidence of side effects [3]. Although 3-4 L of PEG-ELS is used in Western countries, approximately 2 L of PEG-ELS, along with a laxative, is usually considered adequate for bowel preparation in Japan. In our hospital, standard regimen of colonoscopy preparation is a low-residue diet for one day before colonoscopy and 2 L of PEG-ELS with a laxative. Despite the lower-volume in Japan, the need to drink such large volumes of liquid with an unpalatable taste has a negative impact on patient compliance [5]. Therefore, more effective bowel preparation regimens for colonoscopy are needed to improve the acceptability and tolerability of the procedure. In a previous randomized trial, we found that coingestion of 15 mg of Mosapride as a prokinetic was not effective for reducing the volume of PEG-ELS required for bowel preparation [6]. Nevertheless, we found that the 1.5 L PEG-ELS regimen had better patient acceptability and tolerability compared with the 2 L PEG-ELS regimen. Thus the reduction of 0.5 L PEG-ELS volume for bowel preparation is a worthwhile goal, from the patient's perspective.

Recently, a new low-volume hyperosmolar PEG-ELS has become available in Japan. It combines PEG-ELS with a high dose of ascorbic acid (PEG+ASC, Moviprep, Ajinomoto Pharmaceuticals Co., Ltd., Tokyo, Japan). The addition of ascorbic acid reduces the volume of the lavage solution from 4 L to 2 L. The cathartic effects of ascorbic acid are thought to be due to its absorption mechanism that saturates at a high dose [7, 8]. Excess ascorbic acid that cannot be absorbed remains in the bowel where it exerts an osmotic effect, acting synergistically with PEG-ELS. The addition of ascorbic acid also appears to improve the taste of the PEG-ELS preparation. In Western countries, PEG+ASC has been available for bowel preparation for colonoscopy since 2006; and several studies have demonstrated the efficacy, acceptability, tolerability, and safety of PEG+ASC compared with several regimens of standard PEG-ELS [9–20]. In Japan, phase III trial was performed that evaluates the efficacy, acceptability, tolerability, and safety of PEG+ASC compared with standard PEG-ELS without food restriction and laxative. In that study, it took 1.63 ± 0.38 L PEG+ASC to obtain the optimal colonoscopy preparation (not published data). From this result, we suppose that 1.5 L PEG+ASC with food restriction and laxative are equivalent to standard regimen in our hospital.

Because of an ageing population, and increasing burden of colorectal cancer at an advanced age, colonoscopies are increasingly needed in the elderly. Although the efficacy and safety of PEG-ELS have been proven in elderly patients, and those with comorbidities, PEG-ASC has not been evaluated in Japanese patients, particularly those with advanced age.

We therefore designed the present study to compare the efficacy, acceptability, tolerability, and safety of 1.5 L PEG+ASC and 2 L PEG-ELS, not only in the general population, but also in patients of advanced age.

## 2. Patients and Methods

This was a prospective, randomized, controlled, single-center, investigator-blinded, noninferiority study comparing 1.5 L PEG+ASC with 2 L PEG-ELS in patients who underwent colonoscopy. All patients provided written, informed consent. The study was conducted at Aichi Cancer Center Hospital (ACCH), Nagoya, from September 2013 to August 2014. The study was reviewed and approved by the ethics committee of ACCH, and all patients signed an approved informed consent form prior to entering the study. This trial was registered in an international clinical trial registry (UMIN000011505). All consecutive inpatients of both sexes aged 20 years and older who were scheduled for mainly therapeutic colonoscopy such as polypectomy, endoscopic mucosal resection, and endoscopic submucosal dissection at ACCH were evaluated for inclusion in the study. Patients with the following clinical features were excluded: significant cardiac, renal, hepatic, or metabolic comorbidities, ascites, severe constipation (<2 bowel movements per week), known allergy to PEG-ELS, history of gastric stapling or bypass procedure, or history of prior colonic or rectal surgery. Patients were excluded if there was a suspected diagnosis of intestinal obstruction because of advanced colorectal cancer.

### 2.1. Randomization and Blinding

Patients were randomly allocated to receive one of two different bowel preparation regimens using a computer-generated random-number list. Randomization was stratified by age (<70 years or 70≤ years) and performed in blocks of 4. Concealed allocation was accomplished through nonresearch personnel who were not involved in this study. Patients were instructed not to discuss their bowel preparation with anyone other than the unblinded research assistant. With the exceptions of the patient and the unblinded research assistant, all other individuals participating in this study, including the endoscopists and endoscopy nurses, were blinded to the allocated treatment group. Comparisons between the 1.5 L PEG+ASC group and the 2 L PEG-ELS group were made in an investigator-blinded fashion.

### 2.2. Bowel Preparation Methods (Figure 1)

The day before colonoscopy, all patients were admitted to our hospital. An orientation talk was given by a nurse, who carefully explained how the product should be taken, emphasizing the importance of complete intake of the solution to ensure a safe and effective procedure. The subjects were instructed to eat a low-residue diet served in our hospital and asked to drink more than 2 liters of clear liquid. On the evening (up to 22:00) before the day of the colonoscopy, all patients were instructed to take 7.5 mg sodium picosulfate hydrate (Laxoberon: Teijin Pharma, Ltd., Tokyo, Japan). On the day of the colonoscopy, participants received either PEG+ASC (Moviprep: each liter contained 100.0 g macrogol 4000, 7.5 g sodium sulfate, 2.7 g sodium chloride, 1.0 g potassium chloride, 4.7 g ascorbic acid, 5.9 g sodium ascorbate, and lemon flavoring) or PEG-ELS (Niflec: Ajinomoto Pharmaceuticals Co., Ltd., Tokyo, Japan, each liter containing 59.0 g macrogol 4000, 5.7 g sodium sulfate, 1.5 g sodium chloride, 0.7 g potassium chloride, 1.7 g sodium bicarbonate, and lemon flavoring). The PEG-ELS group was instructed to begin drinking 2 L at a rate of 0.25 L every 15 min. The PEG+ASC group was instructed to begin drinking the first 1 L of cleansing solution followed by 0.5 L clear fluid; after that, they were instructed to begin drinking the remaining 0.5 L of cleansing solution followed by 0.25 L clear fluid at a rate of 0.25 L every 15 min. These instructions were in accordance with those of the manufacturer. All patients were instructed to take clear liquids after they finished drinking the cleansing solution. Colonoscopies were scheduled to be performed after 16:00.

### 2.3. Evaluation of Bowel Preparation

The efficacy of the bowel preparation was assessed using the Boston Bowel Preparation Scale (BBPS) [21]. The preparation efficacy was evaluated by the blinded endoscopist per colonic segment (right, transverse, and left colon) on a 4-point scale (0–3) according to the BBPS. In addition, overall cleansing of the colon was scored by summing up the scores of each segment. For the study, the total score of each patient, ranging from 0 to 9, was divided into four different classes: excellent cleansing (total score 8-9), good cleansing (6-7), poor cleansing (3–5), and inadequate cleansing (i.e., requiring additional treatment, 0–2). The participating endoscopists were trained to use the BBPS scale to achieve a good level of agreement. The final assessment of the bowel preparation was divided into two categories, successful and failed. A bowel preparation rated as excellent or good based on the BBPS was considered successful, and poor or inadequate ratings were considered failed. The investigators performed calibration exercises involving more than 20 colonoscopies prior to study commencement, based on their interpretation of scale anchors, to ensure that their findings agreed.

The physicians were also asked to score the overall mucosal visibility according to the following 3-grade scale [22]: optimal (grade 0, clear imaging with no or a minimal amount of bubbles or foam that could be easily removed), adequate (grade 1, modest amount of bubbles and foam that could be cleared with a minimal amount of time), and insufficient (grade 2, presence of foam and bubbles that significantly reduced the clear visualization of the mucosa).

During or immediately following the colonoscopy, the investigator completed a physician questionnaire regarding assessment of the bowel preparation, amount of irrigation fluid used, time needed to reach the cecum, ease of insertion into the cecum, and difficulty in observing the lumen of the colorectum because of peristalsis.

### 2.4. Patient Acceptability, Tolerability, and Other Measurements

The nursing staff recorded the time required to drink the indicated volume of lavage solution. They also recorded the time and number of bowel movements from the start of ingestion to the appearance of clear excretion. Until one hour after finishing the preparation procedure, the nursing staff checked excretions. If there was a solid stool with muddy excretions or no excretion at that time, the patient was given an additional preparation, such as additional PEG-electrolyte solution or enemas. The patients who received an additional preparation were defined by the BBPS as inadequate. The patient questionnaire consisted of 20 questions. Tolerability assessment was based on the recording of GI symptoms such as nausea, vomiting, bloating, and abdominal pain. These events were scored on a 4-point scale: 1 = none, 2 = mild, 3 = moderate, and 4 = severe. The acceptability assessment was based on the willingness to repeat the same preparation regimen. The patients made an entry in the questionnaire form before undergoing colonoscopy and submitted it to the nursing staff.

Blood was sampled at baseline, and 3 hours and 7 hours after the beginning of bowel cleansing for hematological and biochemical analysis. After blood sampling at 7 hours, the scheduled colonoscopy was performed.

### 2.5. End Points

The primary end point was patient acceptability, defined as the rating of “much” willingness to repeat the same preparation regimen. Secondary end points included tolerability, overall colon cleansing (defined as the rate of “successful” cleansing), the rate of optimal mucosa visibility (score 0), and total time for colonic preparation.

### 2.6. Statistical Analysis

Based on a previous study [6], the rate of “much” willingness to repeat the same preparation regimen for the PEG-ELS was expected to be 61.9%. For an adequate rate, we expected about 75% of the PEG+ASC group to give a rating of “much” and the noninferiority margin was set at −10%. This study was designed to have 80% power to establish noninferiority (using a one-sided significance level of 0.025 and a target sample size of 100). If the difference between treatments was above this cutoff but also above the zero difference line, we defined 1.5 L PEG+ASC as superior to 2 L PEG-ELS. The primary analysis for noninferiority was performed on the per protocol (PP) population.

Baseline characteristics were summarized by the usual descriptive statistics, such as the mean and standard deviation for continuous variables and rates for categorical variables. The two-sided *t*-test was used to compare the mean of continuous variables; the likelihood ratio Chi-squared test was used to compare the categorical measures. The paired *t*-test was used to compare the mean of blood data before and after the procedure. *P* values less than 0.05 were considered as statistically significant.

## 3. Results

### 3.1. Patient Characteristics

**Figure 2:**
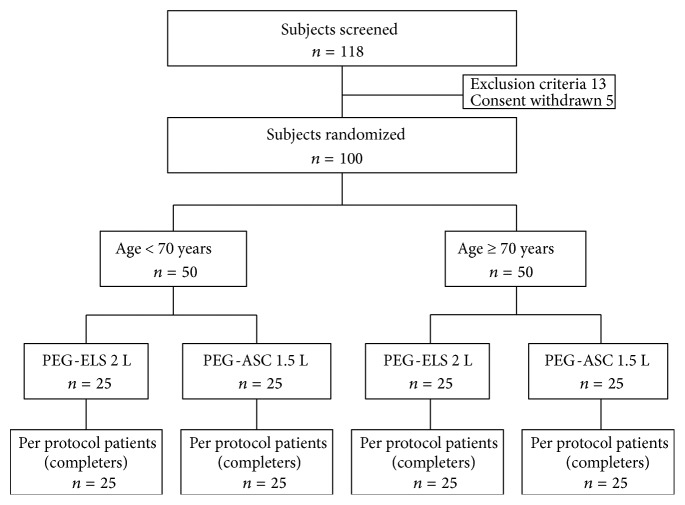
Patient flow.

A total of 100 patients were randomized into two groups (Figure 2). The baseline characteristics of the patients are shown in Table 1. There were no significant differences in age, sex, body mass index, or indications for colonoscopy between the two groups. Ninety-eight of 100 study patients had undergone colonoscopy in our hospital as outpatients in the previous 6 months, at which time colon polyps and/or early colorectal cancer were detected. Therefore, most of the study patients had already experienced bowel preparation with PEG-ELS (Niflec) and colonoscopy in our hospital previously.

### 3.2. Patient Acceptability, Tolerability, and Safety

Patient tolerance and acceptance, as assessed by a questionnaire scoring subjective evaluations, are shown in Table 2. There was no significant difference in compliance between the two groups, as defined by >75% intake of the prescribed bowel cleansing solution volume and complete (100%) intake of the bowel cleansing solution between the two regimens. When asked about their overall impression, the proportion of patients who answered “easy” or “difficult” was significantly different between the two groups (*P* = 0.008). However, symptoms such as nausea, vomiting, bloating, abdominal pain, and circulatory reactions were similar between groups. The primary end point of this study, the rate of patients who declared that they would be willing to repeat the same preparation regimen if needed, was significantly higher in the 1.5 L PEG+ASC group (72%) compared with the 2 L PEG-ELS group (52%). This gave a difference of +20% with a lower limit of the 1-sided 97.5% confidence limit of 1.4% (i.e., within the limits for noninferiority and more superiority set before the study). Furthermore, among the subgroup of 1.5 L PEG+ASC group, who had undergone bowel preparation with 2 L PEG-ELS for colonoscopy within the previous 6 months, 24 of 50 (48%) patients felt that it was easier to ingest 1.5 L PEG+ASC than 2 L PEG-ELS.

### 3.3. Bowel Cleansing Efficacy

The efficacy of bowel preparation is shown in Table 3. There was no significant difference in the successful bowel preparation rate between the 1.5 L PEG+ASC group (92%) and the 2 L PEG-ELS group (82%). The mucosal inspection was rated optimal in 56.4% of the 2 L PEG-ELS group and in 48.0% of the 1.5 L PEG+ASC group (*P* = 0.159). Two patients (4.0%) in each group required additional preparation.

The time to first defecation and the completion time for bowel preparation were significantly shorter in the 1.5 L PEG+ASC group than in the 2 L PEG-ELS group (*P* = 0.041, *P* = 0.030; resp.). There were no differences between the two groups in frequency of defecation, elapsed time from last fluid intake to colonoscopy, amount of irrigation fluid used, time needed for endoscopist to reach the cecum, and subjective difficulties in insertion to the cecum.

### 3.4. Hematological and Biochemical Measurements

Table 4 shows the mean change from screening to 3 hours and to 7 hours after the baseline in both groups. Although many significant fluctuations in hematological and biochemical parameters were noted within both groups, all parameters except sodium bicarbonate at 7 hours after beginning the regimen in the 1.5 L PEG+ASC group changed within the normal range. In the PEG-ELS group, serum albumin, alanine aminotransferase (ALT), and pH were significantly increased at both 3 hours and 7 hours. Serum glucose, sodium, chloride, phosphorus, magnesium, and osmolarity were significantly decreased at both 3 hours and 7 hours. In the 1.5 L PEG+ASC group, serum total protein, albumin, ALT, red blood cells, and hematocrit were significantly increased at both 3 hours and 7 hours. Serum sodium, chloride, pH, sodium bicarbonate, base excess, and osmolarity were significantly decreased at both 3 hours and 7 hours.

Comparison of hematological and biochemical changes in patients under 70 years of age is shown in Table 5. Regarding parameter differences between 3 hours and the beginning, the differences in the serum creatinine, chloride, and magnesium values were significantly larger in the 2 L PEG-ELS group, and the differences in the serum phosphorus, pH, sodium bicarbonate, and base excess values were significantly larger in the 1.5 L PEG+ASC group. Regarding the differences between 7 hours and the beginning, the differences in the blood urea nitrogen (BUN), serum potassium, chloride, and phosphorus values were significantly larger in the 2 L PEG-ELS group, and the differences in the pH, sodium bicarbonate, base excess, red blood cell, and hematocrit values were significantly larger in the 1.5 L PEG+ASC group.

Comparison of hematological and biochemical changes in patients aged 70 years and over is shown in Table 6. Regarding the differences between 3 hours and the beginning, the differences in the serum chloride and magnesium values were significantly larger in the 2 L PEG-ELS group, and the differences in the serum creatinine, phosphorus, pH, sodium bicarbonate, and base excess values were significantly larger in the 1.5 L PEG+ASC group. Regarding the differences between 7 hours and the beginning, the differences in the chloride and phosphorus values were significantly larger in the 2 L PEG-ELS group, and the differences in the pH, sodium bicarbonate, and base excess values were significantly larger in the 1.5 L PEG+ASC group.

There were no statistically or clinically significant differences between subjects under or over 70 years of age in hematological and biochemical changes between before and after taking 2 L PEG-ELS or 1.5 L PEG+ASC.

## 4. Discussion

Colonoscopy is considered the most effective procedure for early detection and prevention of colorectal cancer [23, 24]. Adequate bowel preparation is essential for an effective colonoscopy evaluation [1]. Inadequate bowel preparation can result in missed lesions, aborted procedures, and increased discomfort as well as a potential increase in complication rates [25–28]. Large-volume PEG-ELS preparations are used mainly because of their favorable safety profile and proven efficacy so far [2, 3]. However, the major limitation of their use is the volume of preparation to be ingested, which may have a negative impact on patient acceptability, compliance, and, as a result, reduction in overall efficacy [26, 27].

Several low-volume regimens have been introduced into clinical practice. They are based on the combination of low-volume PEG-ELS with a stimulant laxative—senna or bisacodyl [29, 30] or a prokinetic—mosapride or itopride [31, 32]. These low-volume regimens have shown similar efficacy and higher acceptability than the standard dose of PEG-ELS [29–32]. Current trends are leading towards an increase in the use of low-volume preparations, and the combination of 2 L PEG+ASC is being considered as a market leader in the Western countries. Recently, PEG+ASC has become available in Japan. Although the efficacy, acceptability, tolerability, and safety of PEG+ASC compared with standard-volume PEG-ELS have already been demonstrated in the Western countries, PEG+ASC has never been evaluated in Japanese patients.

The present study demonstrated that 1.5 L PEG+ASC solution was not inferior, but instead superior to 2 L PEG-ELS in patient acceptability of bowel preparation for colonoscopy. At the same time, 1.5 L PEG+ASC was similar to 2 L PEG-ELS in bowel cleansing efficacy, tolerability, and safety. These results are in line with previous studies [9–20] that compared the efficacy, acceptability, tolerability, and safety of low-volume PEG+ASC and standard-volume PEG-ELS for bowel preparation for colonoscopy. Ell et al. [9] showed that 2 L PEG+ASC was not inferior to 4 L PEG-ELS in bowel cleansing, with better acceptability of 2 L PEG+ASC than 4 L PEG-ELS in an inpatient setting, using a split-dose regimen the evening before colonoscopy and the following morning. Ponchon et al. [17] showed that 2 L PEG+ASC produced a similar high degree of cleansing and superior acceptability and tolerability compared with 4 L PEG-ELS in an outpatient setting, using a single-dose regimen the evening before colonoscopy. Indeed, there was heterogeneity among previous trials [9–20] regarding variations in the timing of bowel preparation, in the dosage schedule, in dietary instruction prior to and during the preparation, in diverse uses of the bowel preparation scale, and in the use of different adjuvants; these may all have contributed to those results. However, recently, Xie et al. [33] reported a meta-analysis of randomized controlled trials of low-volume PEG+ASC versus standard-volume PEG-ELS as bowel preparations for colonoscopy. In this report, eleven randomized controlled trials were identified for analysis; Xie et al. [33] demonstrated that the low-volume PEG+ASC achieved noninferior efficacy for bowel cleansing, was more acceptable to patients, and produced fewer side effects than the standard-volume PEG-ELS.

The present study showed no significant difference in bowel cleansing efficacy between the PEG+ASC group and the PEG-ELS group. However, the use of irrigation fluid seemed to be more frequent in the PEG+ASC group than in the PEG-ELS group. One of the reasons may have been foam and bubbles in the colonic lumen. In the present study, we evaluated the overall mucosal visibility that defined the amount of bubbles and foam. Although there were no significant differences between both groups, bubbles and foam may influence the results of the use of irrigation fluid. Several PEG-ELS formulations have added simethicone, which is an oral antifoaming agent that decreases bloating, abdominal discomfort, and abdominal pain by promoting the clearance of excessive gas along the gastrointestinal tract by reducing the surface tension of air bubbles [14, 22]. The combination of simethicone with PEG+ASC may be one of the choices for improving the efficacy, acceptability, and tolerability of PEG+ASC.

Patients favor preparations that are low in volume, are palatable, and have easy-to-complete regimens. However, contrary to our expectations, the present study found no significant difference in the comparison of taste between the two regimens, in contrast to the results of a previous study [9, 17]. One of the reasons is that most of this study's patients had already experienced bowel preparation of 2 L PEG-ELS in the previous 6 months, and they could get used to the flavor of PEG-ELS. Another reason is that patient compliance and acceptability may not be dependent on palatability, but rather the amount of PEG-ELS. In the present study, the reduction volume of PEG-ELS was only 0.5 L. However, there was much difference in the reduction volume as 2 L in previous studies [9–11, 13–20]. The reduction of the volume of PEG-ELS, regardless of the dose, despite being partly compensated by additional PEG-free clear liquid, must lead to improvement in patient acceptability of the bowel preparation. Better compliance, combined with the laxative effect of ascorbic acid, may account for the similar bowel preparation efficacies between the lower-volume PEG-ELS and the standard-volume PEG-ELS.

We demonstrated that the time to first defecation and the completion time for bowel preparation were significantly shorter in the 1.5 L PEG-ASC group than in the 2 L PEG-ELS group. Although the differences of shorted times are 15 minutes and 22 minutes, respectively, these saving times may lead to the improvement of patient acceptability in PEG-ASC group. Furthermore, these results will also provide a merit in saving time for the medical staffs. However, they should pay attention to patients because some patients in PEG-ASC group feel a need to evacuate their bowels rapidly compared to patients in PEG-ELS group.

PEG-ELS has been used worldwide since 1980 because of its well-established safety profile [4, 34, 35]. Because PEG-ELS is isotonic and electrolyte-balanced, there is little change in patient hydration and electrolytes in spite of the large volumes required [4, 34, 35]. On the other hand, although ascorbic acid is known to be safe even when taken in large doses [36, 37], there is concern about the occurrence of dehydration and electrolyte disturbance during bowel preparation with PEG+ASC compared with PEG-ELS, especially in elderly patients, because ascorbic acid possesses cathartic activity. Therefore, we evaluated standard biochemical and hematological parameters before starting the preparation (baseline) and at 3 hrs and 7 hrs after baseline. The usual time period to perform colonoscopy is seven hours or more, after starting the ingestion of the lavage solution. In our hospital, conventional colonoscopy for outpatients is conducted in the afternoon, with morning bowel cleansing. After finishing the outpatient colonoscopies, we prepare for endoscopic treatment of inpatients. During the waiting time after bowel preparation, we encourage patients to drink clear liquids to prevent dehydration. In our hospital, all endoscopic treatments for colorectal neoplasms are planned with admission the day before endoscopic treatment.

It was notable how many biochemical and hematological parameters had changed significantly after ingestion of the cleansing solutions, not only with PEG+ASC but also with PEG-ELS. Most of these changes continued until 7 hours after taking the bowel preparation. However, with the exception of sodium bicarbonate in the PEG+ASC group, all hematological and biochemical parameters varied within the normal range. The addition of electrolytes and ascorbic acid to high molecular weight PEG-ELS may compensate for those changes, reducing the risk of electrolyte disturbances that can occur with other types of bowel preparation. However, there were distinctive biochemical changes in both groups. In the 2 L PEG-ELS group, electrolytes such as sodium and chloride, as well as glucose, had a tendency to decrease compared with the 1.5 L PEG+ASC group. On the other hand, in the 1.5 L PEG+ASC group, dehydration parameters such as total protein, albumin, and hematocrit had a tendency to increase, and acid-base balance parameters such as pH, sodium bicarbonate, base excess, and plasma osmolality had a tendency to decrease compared with the 2 L PEG-ELS group. Particularly, the sodium bicarbonate value in the 1.5 L PEG+ASC group decreased to beyond the lower normal range. These significant changes might be related to the composition of 2 L PEG-ELS and 1.5 L PEG+ASC. PEG+ASC contains ascorbic acid and sodium ascorbate instead of sodium sulfate that is present in PEG-ELS. These differences in composition produce hypertonia and higher acidity in PEG+ACS compared with PEG-ELS.

We also evaluated the differences in hematological and biochemical parameters between subjects younger and older than 70 years. There were no significant differences between the two age groups in any parameters before and after taking PEG-ELS and PEG+ASC. In both age groups, there was a tendency toward dehydration in the 1.5 L PEG+ASC group compared with the 2 L PEG-ELS group, showing that adequate hydration is needed. Although iso-osmotically balanced, PEG-ELS nevertheless has the ability in rare patients to induce hypovolemia combined with dysnatremia due to diarrhea, vomiting, and inadequate hydration during preparation. Hyponatremic hypovolemia can occur when PEG-ELS-induced volume loss results in an upregulation of arginine vasopressin, causing the patient to retain more free water than sodium [38]. When patients are unable to compensate for intestinal losses (such as the elderly with diminished thirst sensation) hypernatremic hypovolemia can occur [39]. Needless to say, the same warning is needed when using PEG+ASC.

The key strength of this study lies in its design. Randomization was stratified by age (<70 years or ≥70 years), and hematological and biochemical parameters were compared using blood samples in each age group, especially with respect to the safety profile of each regimen. We demonstrated significant statistical changes in the hematological and biochemical parameters after both types of preparation regardless of patient age, which were not clinically significant.

There are several limitations to consider in interpreting the results of this study. First, it was conducted in a single hospital with a small number of patients, that is, in inpatients scheduled to undergo endoscopic treatment. We obtained blood samples at baseline, and 3 hours and 7 hours after the patients took the preparation solution. Second, this study was designed to be conducted in an inpatient setting to maximize compliance. These features were incorporated to minimize bias and give high rates of good bowel preparation in both groups. The patients received their bowel preparation from a nurse who supervised its consumption, which probably provided a more accurate measure of compliance than when patients take the preparation at home. Therefore, the compliance rates with the bowel preparation solution in this study are probably higher than those normally achieved in routine outpatient use. Third, most of this study's patients had already experienced bowel preparation of 2 L PEG-ELS in the previous 6 months that may favor PEG+ASC as a new method and may lead to better acceptability in PEG-ASC. Fourth, we evaluated the efficacy, acceptability, tolerability, and safety of 1.5 L PEG+ASC compared with 2 L PEG-ELS; the results may not be applicable to patients in Western Hemisphere because 3-4 L of PEG-ELS has been used in there. Finally, we could not record any additional fluid intake during colonoscopy preparation, which may have influenced the grading of tolerance and acceptability. However, we do not believe that the additional fluid consumption contributed significantly to the efficacy in each group, as much of the excess fluid is absorbed in the upper gastrointestinal tract and excreted via the urinary system. Ell et al. [9] showed that the bowel cleaning efficacy is unrelated to additional fluid ingestion.

In conclusion, the present study demonstrated that patient acceptability was superior with the 1–5 L PEG+ASC regimen than with the 2 L PEG-ELS regimen; however, tolerability, safety, and bowel cleansing were similar in both groups. No significant differences in the safety profile were found between subjects aged less than 70 years and those aged 70 years or more; nevertheless, regardless of age, proper hydration is needed throughout the bowel preparation process.

## Figures and Tables

**Figure 1:**
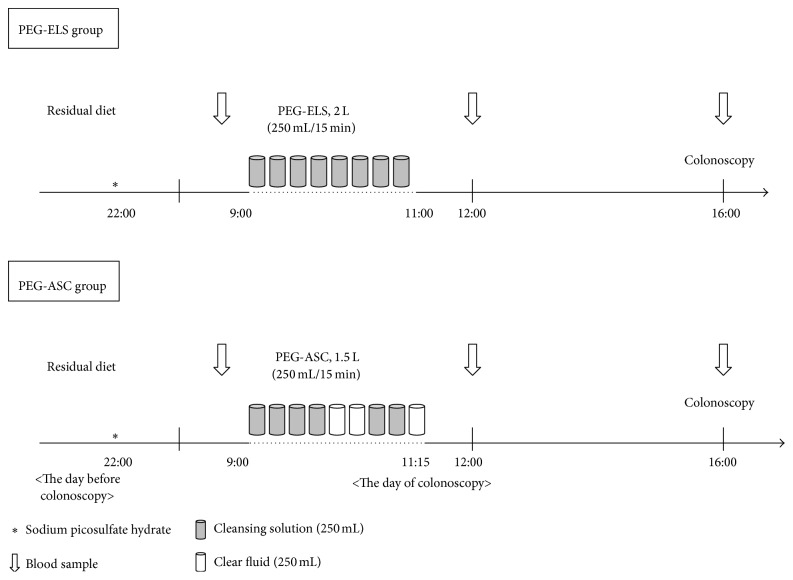
The study schedule. PEG-ELS, polyethylene glycol electrolyte lavage solution. PEG-ASC, polyethylene glycol electrolyte lavage solution containing ascorbic acid.

**Table 1:** Patient characteristics.

Variable	PEG-ELS	PEG-ASC	*P* value
Number of patients	50	50	
<70/≥70	25/25	25/25	1^∗^
Age (years)			
mean ± SD	67.9 ± 9.4	64.6 ± 13.4	0.150^∗∗^
median (range)	69.5 (35–83)	68.5 (27–89)	0.841^∗^
<70; mean ± SD	57.4 + 9.5	56.9 + 10.3	0.842^∗∗^
≥70; mean ± SD	74.8 + 3.6	75.5 + 5.0	0.559^∗∗^
Male/female	32/18	33/17	0.834^∗^
<70	17/8	17/8	1^∗^
≥70	15/10	16/9	0.770^∗^
Body mass index (kg/m^2^)	22.8 + 3.8	22.8 + 4.1	0.982^∗∗^
Reason for colonoscopy, *n*			
EMR/ESD	49	50	1^∗^
Anemia and advanced age	1	0	

^∗^Chi-square test; ^∗∗^Welch's test. EMR, endoscopic mucosal resection; ESD, endoscopic submucosal dissection.

**Table 2:** Patient tolerance and acceptance.

Variable	PEG-ELS	PEG-ASC	*P* value
Number of patients	50	50	
Compliance >75% (*n*, %)	45 (90)	47 (94)	0.461^∗^
100% intake (*n*, %)	44 (88)	44 (88)	1^∗^
How was the taste of preparation liquid?			
(very good/good/fair/bad/unacceptable)	(0/3/28/17/2)	(0/9/27/13/1)	0.099^∗∗^
At what volume did you feel distress?			
(<500/<1000/<1500/<2000/no distress)	(3/12/21/5/9)	(6/15/15/0/14)	0.537^∗∗^
How easy/difficult to take preparation (overall impression)?			
(very easy/easy/fair/difficult/very difficult)	(1/4/24/15/6)	(1/16/20/11/2)	0.008^∗∗^
Any symptoms (*n*)			
Nausea			
(none/mild/moderate/severe)	(42/4/2/2)	(41/6/3/0)	0.870^∗∗^
Vomiting			
(no/yes)	(48/2)	(49/1)	0.558^∗^
Distension			
(none/mild/moderate/severe)	(8/22/18/2)	(16/21/9/4)	0.079^∗∗^
Abdominal pain			
(none/mild/moderate/severe)	(43/5/2/0)	(43/6/1/0)	0.973^∗∗^
Circulatory reactions			
(none/mild/moderate/severe)	0	0	1^∗∗^
Willingness to repeat (*n*) the same preparation regimen			
(much/somewhat/never)	(26/9/15)	(36/7/7)	0.031^∗∗^
Willingness to repeat (rate of much, %)	52	72	**0.039**
Point estimate and 95% CI the difference between for willingness to repeat	**+20 (0.014, 0.386)**		
How easy/difficult to undergo this preparation compared with previous one, PEG (*n*)			
(easy/intermediate/difficult)		(24/19/7)	

PEG, polyethylene glycol; ASC, ascorbic acid; N.S., not significant.

^∗^Chi-square test, ^∗∗^Mann-Whitney *U* test.

**Table 3:** Results of the preparation and endoscopic findings.

Variable	PEG-ELS	PEG-ASC	*P* value
Number of patients	50	50	
Time to first defecation (min, mean ± SD)	64 ± 41	49 ± 33	0.041^∗^
Frequency of defecation (times, median, quartile)	8 (3–31)	7 (3–15)	0.160^∗∗^
Time to preparation (min, mean ± SD)	194 ± 69	172 ± 77	0.030^∗^
Elapsed time from last fluid intake to colonoscopy (min, mean ± SD)	217 ± 80	238 ± 81	0.181^∗^
Cecal intubation rate (*n*, %)	50 (100)	50 (100)	1.000^∗∗∗^
Insertion time (min, median, quartile)^∗^	7.5 (3–30)	7 (3–21)	0.689^∗^
Feel of peristalsis (*n*, %)	9 (18)	10 (20)	0.799^∗∗∗^
Qualitative preparation rating (*n*, %)			
Excellent	24	22	
Good	17	24	0.889^∗∗^
Poor	7	2	
Inadequate (additional treatment)	2	2	
Successful bowel cleansing (*n*, %)	41 (82)	46 (92)	0.234^∗∗∗^
Optimum visibility grade (0/1/2)	31/14/5	24/17/9	0.133^∗∗^
Amount of irrigation fluid (*n*)			
None	10	8	
≤100 mL	32	24	0.063^∗∗^
100 mL<	8	18	

^∗^Welch test; ^∗∗^Mann-Whitney *U* test; ^∗∗∗^Chi-square test.

**Table 4:** Laboratory results comparing baseline to 3 hours and 7 hours after taking the gut lavage solution.

		PEG-ELS			*P* value		PEG-ASC			*P* value	
		I	II	III	I vs II	I vs III	I	II	III	I vs II	I vs III
Total protein (g/dL)	6.7–8.3	6.9	7.0	7.0	0.086	0.223	6.9	7.2	7.1	**<0.001**	**<0.001**
Albumin (g/dL)	4.0–5.0	4.2	4.3	4.3	0.015	0.004	4.2	4.4	4.4	**<0.001**	**<0.001**
Glucose (mg/dL)	70–109	100.0	92.2	92.0	0.002	<0.001	93.1	89.5	90.6	0.076	0.320
BUN (mg/dL)	8–22	13.5	14.7	12.8	0.513	<0.001	13.3	13.3	13.5	0.917	0.441
Creatinine (mg/dL)	0.60–1.10	0.74	0.72	0.73	0.077	0.451	0.74	0.76	0.74	**0.003**	0.344
ALT (U/L)	6–30	23.4	26.0	25.4	**<0.001**	**<0.001**	23.4	26.7	26.9	**<0.001**	**<0.001**
Sodium (mmol/L)	138–146	140.4	139.7	139.5	**0.015**	**0.006**	140.5	139.9	139.1	**0.033**	**<0.001**
Potassium (mmol/L)	3.6–4.9	4.2	4.1	3.9	0.264	**<0.001**	4.2	4.3	4.1	**0.009**	**0.004**
Chloride (mmol/L)	99–109	105.1	103.5	102.9	**<0.001**	**<0.001**	105.1	105.9	104.9	**0.003**	0.422
Calcium (mg/dL)	8.7–10.3	9.1	9.0	9.0	0.148	0.189	9.1	9.2	9.2	0.082	0.665
Phosphorus (mg/dL)	2.5–4.7	3.5	3.5	3.3	**0.086**	**<0.001**	3.5	3.8	3.4	**<0.001**	0.752
pH	7.32–7.42	7.38	7.39	7.39	**0.001**	**0.005**	7.38	7.35	7.35	**<0.001**	**<0.001**
Sodium bicarbonate	24.0–28.0	27.5	27.4	26.8	0.802	0.062	28.1	24.2	23.8	**<0.001**	**<0.001**
Base excess (mmol/L)	−2.5–2.5	1.6	1.8	1.4	0.331	0.415	2.1	−1.7	−2.0	**<0.001**	**<0.001**
Osmolality (mOsm/kg)	285–295	291.2	289.8	288.6	**0.059**	**<0.001**	290.9	289.6	288.1	**0.011**	**<0.001**
Magnesium (mg/dL)	1.8–2.4	2.1	2.0	2.0	**<0.001**	**<0.001**	2.0	2.1	2.0	0.141	**<0.001**
White blood cells (/*μ*L)	3400–8400	5622.8	5853.6	6732.8	0.129	**<0.001**	5753.2	5807.4	7473.4	0.636	**<0.001**
Red blood cells (×10^4^/*μ*L)	420–560	431.2	424.5	430.3	0.306	0.729	446.3	455.7	454.2	**0.001**	**0.002**
Hemoglobin (g/dL)	M: 13.5–18.0	13.6	13.5	13.5	0.363	0.171	13.9	14.0	14.6	0.912	0.261
	F: 11.3–14.9										
Hematocrit (%)	M: 39.9–50.3	40.9	40.7	40.6	0.500	0.304	41.6	42.7	42.5	**0.001**	**0.005**
	F: 34.8–44.0										
Neutrophils (%)		58.3	60.1	59.0	**0.039**	0.522	59.0	62.8	63.4	**0.001**	**<0.001**
Lymphocytes (%)		30.7	29.7	31.6	0.142	0.236	30.7	27.9	28.5	**0.005**	**0.032**
Eosinophils (%)		2.7	2.1	1.8	**<0.001**	**<0.001**	2.8	2.1	1.7	**<0.001**	**<0.001**

I, before preparation; II, 3 hrs after beginning of preparation; III, 7 hrs after beginning of preparation; *P* value by paired *t*-test two-side. BUN, blood urea nitrogen; ALT, alanine aminotransferase.

**Table 5:** Differences in laboratory results between baseline and 3 hours and 7 hours after taking gut lavage solution in patients aged less than 70 years.

	Difference between 3 hrs and baseline			Difference between 7 hrs and baseline		
	PEG-ELS	PEG-ASC	*P*-value	PEG-ELS	PEG-ASC	*P*-value
Total protein (g/dL)	0.1 (0.4)	0.4 (0.4)	0.013	0.0 (0.4)	0.2 (0.3)	0.068
Albumin (g/dL)	0.1 (0.2)	0.3 (0.3)	0.044	0.1 (0.3)	0.2 (0.2)	0.152
Glucose (mg/dL)	−5.2 (9.7)	−2.9 (6.8)	0.325	−11.1 (19.6)	−6.4 (8.1)	0.279
BUN (mg/dL)	−0.6 (1.2)	0.1 (1.5)	0.072	−0.9 (1.3)	0.0 (1.5)	**0.023**
Creatinine (mg/dL)	−0.02 (0.05)	0.01 (0.05)	**0.030**	−0.02 (0.05)	0.02 (0.05)	**0.025**
ALT (U/L)	2.5 (2.6)	3.4 (2.9)	0.218	2.2 (2.9)	3.3 (3.4)	0.202
Sodium (mmol/L)	−0.8 (2.4)	−0.7 (2.2)	0.903	−1.2 (2.7)	−1.3 (1.6)	0.901
Potassium (mmol/L)	0.0 (0.5)	0.1 (0.2)	0.117	−0.3 (0.3)	−0.1 (0.3)	**0.017**
Chloride (mmol/L)	−1.5 (2.3)	0.6 (1.9)	**<0.001**	−2.1 (2.8)	−0.1 (1.9)	**0.006**
Calcium (mg/dL)	0.0 (0.3)	0.1 (0.2)	0.186	0.0 (0.4)	0.1 (0.2)	0.269
Phosphorus (mg/dL)	−0.1 (0.2)	0.3 (0.3)	**<0.001**	−0.3 (0.3)	0.0 (0.3)	**<0.001**
pH	0.02 (0.03)	−0.04 (0.04)	**<0.001**	0.02 (0.03)	−0.04 (0.04)	**<0.001**
Sodium bicarbonate	−0.3 (2.7)	−4.0 (2.6)	**<0.001**	−0.8 (2.4)	−4.4 (2.6)	**<0.001**
Base excess (mmol/L)	0.1 (2.1)	−4.0 (1.9)	**<0.001**	−0.3 (1.8)	−4.4 (2.1)	**<0.001**
Osmolality (mOsm/kg)	−2.1 (4.6)	−1.6 (4.4)	0.669	−3.3 (5.1)	−2.9 (3.6)	0.732
Magnesium (mg/dL)	−0.1 (0.1)	0.0 (0.1)	**<0.001**	−0.1 (0.1)	−0.1 (0.1)	0.120
White blood cells (/*μ*L)	230 (725)	261 (784)	0.884	997 (1809)	1951 (1869)	0.073
Red blood cells (×10^4^/*μ*L)	−12.5 (62.4)	13.7 (17.0)	0.052	−1.7 (18.1)	8.2 (13.9)	**0.035**
Hemoglobin (g/dL)	−0.1 (0.5)	0.0 (2.1)	0.896	−0.1 (0.6)	1.2 (5.4)	0.234
Hematocrit (%)	−0.2 (1.6)	1.3 (1.7)	**0.003**	−0.4 (1.9)	0.8 (1.4)	**0.013**
Neutrophils (%)	1.3 (5.9)	3.0 (8.3)	0.404	2.1 (7.6)	6.2 (10.0)	0.107
Lymphocytes (%)	−0.7 (4.4)	−2.0 (7.3)	0.432	−0.7 (5.7)	−4.0 (8.4)	0.108
Eosinophils (%)	−0.6 (0.6)	−0.8 (1.1)	0.367	−1.0 (0..8)	−1.3 (1.6)	0.422

Values are mean (SD), *P* value by Welch's *t*-test, two-side.

**Table 6:** Differences in laboratory results between baseline and 3 hours and 7 hours after taking gut lavage solution in patients aged 70 years or more.

	Difference between 3 hrs and baseline			Difference between 7 hrs and baseline		
	PEG-ELS	PEG-ASC	*P*-value	PEG-ELS	PEG-ASC	*P*-value
Total protein (g/dL)	0.1 (0.4)	0.2 (0.5)	0.283	0.1 (0.3)	0.2 (0.4)	0.414
Albumin (g/dL)	0.1 (0.3)	0.1 (0.3)	0.420	0.1 (0.2)	0.1 (0.3)	0.817
Glucose (mg/dL)	−6.4 (8.4)	−4.4 (19.0)	0.627	−8.6 (16.6)	1.4 (23.1)	0.085
BUN (mg/dL)	−0.5 (1.1)	0.0 (1.2)	0.146	−0.7 (1.8)	0.3 (1.8)	0.055
Creatinine (mg/dL)	−0.01 (0.06)	0.03 (0.05)	**0.009**	0.00 (0.09)	0.00 (0.05)	0.828
ALT (U/L)	2.4 (2.0)	3.2 (5.3)	0.485	1.8 (1.9)	3.7 (6.1)	0.152
Sodium (mmol/L)	−0.6 (1.6)	−0.4 (1.4)	0.642	−0.7 (1.9)	−1.4 (1.9)	0.194
Potassium (mmol/L)	−0.1 (0.3)	0.0 (0.2)	0.070	−0.3 (0.3)	−0.2 (0.3)	0.130
Chloride (mmol/L)	−1.6 (1.3)	0.8 (1.4)	**<0.001**	−2.3 (2.2)	−0.3 (2.0)	**0.001**
Calcium (mg/dL)	−0.1 (0.3)	0.0 (0.4)	0.064	−0.1 (0.4)	0.0 (0.3)	0.417
Phosphorus (mg/dL)	0.0 (0.2)	0.3 (0.2)	**<0.001**	−0.2 (0.3)	0.0 (0.2)	**0.002**
pH	0.01 (0.03)	−0.03 (0.03)	**<0.001**	0.01 (0.03)	−0.02 (0.03)	**0.004**
Sodium bicarbonate	0.1 (2.5)	−3.9 (2.2)	**<0.001**	−0.4 (2.1)	−4.3 (2.1)	**<0.001**
Base excess (mmol/L)	0.4 (1.8)	−3.7 (2.1)	**<0.001**	−0.1 (1.7)	−3.9 (2.1)	**<0.001**
Osmolality (mOsm/kg)	−0.8 (6.1)	−1.1 (2.8)	0.815	−2.2 (3.9)	−2.7 (4.1)	0.643
Magnesium (mg/dL)	−0.1 (0.1)	0.0 (0.1)	**0.002**	−0.1 (0.1)	−0.1 (0.1)	0.787
White blood cells (/*μ*L)	231 (1327)	−153 (789)	0.220	1223 (1024)	1489 (1021)	0.361
Red blood cells (×10^4^/*μ*L)	−0.9 (17.4)	5.2 (21.3)	0.277	−0.2 (19.6)	7.5 (19.6)	0.175
Hemoglobin (g/dL)	−0.1 (0.6)	0.0 (0.7)	0.458	−0.1 (0.6)	0.0 (0.6)	0.421
Hematocrit (%)	−0.1 (1.7)	0.9 (2.5)	0.103	−0.1 (2.0)	1.0 (2.7)	0.105
Neutrophils (%)	2.2 (5.8)	4.5 (7.0)	0.203	−0.8 (5.8)	2.6 (6.3)	0.052
Lymphocytes (%)	−1.2 (4.8)	−3.6 (6.3)	0.151	2.5 (4.7)	−0.4 (5.0)	**0.037**
Eosinophils (%)	−0.7 (0.7)	−0.6 (0.7)	0.621	−0.9 (1.0)	−1.0 (1.0)	0.669

Values are mean (SD), *P* value by Welch's *t*-test, two-side.
